# *ASXL1* mutation as a surrogate marker in acute myeloid leukemia with myelodysplasia‐related changes and normal karyotype

**DOI:** 10.1002/cam4.2947

**Published:** 2020-03-26

**Authors:** Concepción Prats‐Martín, Sergio Burillo‐Sanz, Rosario M. Morales‐Camacho, Olga Pérez‐López, Milagros Suito, Maria T. Vargas, Teresa Caballero‐Velázquez, Estrella Carrillo‐Cruz, José González, Ricardo Bernal, José A. Pérez‐Simón

**Affiliations:** ^1^ Department of Hematology Hospital Universitario Virgen del Rocío Instituto de Biomedicina de Sevilla (IBIS/CISC/CIBERONC) Universidad de Sevilla Sevilla Spain; ^2^ Department of Immunology Hospital Universitario Virgen del Rocío. Sevilla Sevilla Spain; ^3^ Department of Hematology Hospital Universitario Virgen Macarena Universidad de Sevilla Sevilla Spain

**Keywords:** AML‐MRC, *ASXL1*, myelodysplasia, myeloid leukemia

## Abstract

Acute myeloid leukemia with myelodysplasia‐related changes (AML‐MRC) are poor outcome leukemias. Its diagnosis is based on clinical, cytogenetic, and cytomorphologic criteria, last criterion being sometimes difficult to assess. A high frequency of *ASXL1* mutations have been described in this leukemia. We sequenced *ASXL1* gene mutations in 61 patients with AML‐MRC and 46 controls with acute myeloid leukemia without other specifications (AML‐NOS) to identify clinical, cytomorphologic, and cytogenetic characteristics associated with *ASXL1* mutational status. Mutated *ASXL1* (*ASXL1+*) was observed in 31% of patients with AML‐MRC compared to 4.3% in AML‐NOS. Its presence in AML‐MRC was associated with older age, a previous history of myelodysplastic syndrome (MDS) or myelodysplastic/myeloproliferative neoplasms (MDS/MPN), leukocytosis, presence of micromegakaryocytes in bone marrow, lower number of blasts in bone marrow, myelomonocytic/monocytic morphological features and normal karyotype. *ASXL1* mutation was not observed in patients with myelodysplastic syndrome‐related cytogenetic abnormalities or *TP53* mutations. Differences in terms of overall survival were found only in AML‐MRC patients without prior MDS or MDS/MPN and with intermediate‐risk karyotype, having *ASXL1+* patients a worst outcome than *ASXL1−*. We conclude that the *ASXL1* mutation frequency is high in AML‐MRC patients being its presence associated with specific characteristics including morphological signs of dysplasia. This association raises the possible role of *ASXL1* as a surrogate marker in AML‐MRC, which could facilitate the diagnosis of patients within this group when the karyotype is normal, and especially when the assessment of multilineage dysplasia morphologically is difficult. This mutation could be used as a worst outcome marker in de novo AML‐MRC with intermediate‐risk karyotype.

## Graphical abstract

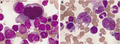

## 1 INTRODUCTION

AML‐MRC are a frequent subtype of leukemia that have poor prognosis.^1^, ^2^, ^3^ Diagnosis is based on the presence of blasts in a percentage equal to or greater than 20% in peripheral blood or bone marrow, associated with morphological features of dysplasia, and/or previous history of MDS or MDS/MPN, and/or presence of MDS‐related cytogenetic abnormalities.^4^ Cases of acute myeloid leukemia (AML) with recurrent genetic abnormalities and therapy‐related AML are excluded. Likewise, since the last update of the World Health Organization (WHO) classification, cases with biallelic *CEBPA* mutation or *NPM1* mutation, in the absence of cytogenetic abnormalities diagnostic of AML‐MRC or a prior history of MDS or MDS/MPN, have been excluded from this group.^5^, ^6^, ^7^ Numerous studies have questioned the independent predictive value of myelodysplasia in the absence of high‐risk cytogenetic abnormalities in AML.^8^, ^9^, ^10^, ^11^, ^12^ It has also been discussed whether a more restrictive definition of the multilineage dysplasia criteria established by WHO or the consideration of only certain specific features of dysplasia (micromegakaryocytes and hypogranulated neutrophils), could better categorize patients in this AML‐MRC group.^13^ Even so, the detection of dysplasia has shown to differentiate patients with an adverse prognosis, excluding cases of AML with *NPM1* or biallelic *CEBPA* mutation.^1^, ^2^, ^6^, ^14^

In recent years, the use of sequencing gene panels has allowed to evaluate the presence of mutations in myeloid neoplasms. Its application in AML‐MRC could contribute to diagnosis, especially in those patients with absence of cytogenetic risk abnormalities or history of MDS or MDS/MPN. In AML‐MRC, mutations in the following genes are found: *ASXL1* (21%‐35%), *TP53* (22%), *RUNX1* (15%‐17%), *TET2* (15%), *IDH1* or *IDH2* (25%), *DNMT3A* (8%‐9%), *NPM1* (8%), and *FLT3* (2%‐7%). From those, only *ASXL1* loss of function mutations and *TP53* mutations showed a prognostic significance.^15^, ^16^

In MDS, loss‐of‐function mutations were found in the *ASXL1* gene by aCGH analysis.^17^ These are typically *nonsense* or *frame‐shift* mutations in heterozygosis in the last exon of the gene, which truncate the protein before the C‐terminal PHD domain, resulting in a haploinsufficiency or a dominant negative effect. The PHD domain, which is truncated in mutated *ASXL1*, can bind to methylated lysins, and interact with the PRC2 complex, implicated in the addition of repressive H3K27me3 marks. Therefore, the inhibition of *ASXL1* leads to the loss of recruitment of PRC2 and thus to the loss of repressive histones in leukemogenic loci, such as the *HOXA* cluster, which leads to a higher expression of the *HOXA5‐9* genes. These and other data suggest a role for the loss of *ASXL1* in leukemogenesis.^18^ In this sense, these mutations have been associated with a shorter time of transformation from MDS and chronic myelomonocytic leukemia (CMML) to AML.^19^, ^20^, ^21^ The most prevalent mutation in myeloid neoplasms is p.G646Wfs*12, which is currently considered a true pathogenic mutation.^20^, ^21^, ^22^ A prognostic value of mutations in *ASXL1* has been demonstrated in MDS,^20^ CMML,^19^ myeloproliferative neoplasms (MPN) 22 and primary myelofibrosis (PMF).^23^ In AML, mutations in *ASXL1* have been associated with an adverse outcome in patients with intermediate‐risk karyotypes.^24^ *ASXL1* mutations have been reported in up to 35% of patients with intermediate‐risk and in 5% of unfavorable‐risk karyotypes AML‐MRC. They can occur simultaneously with *RUNX1* and *FLT3* mutations but have not been described together with *NPM1* mutations.^16^ Previously, association of *ASXL1* to AML‐MRC has been described.^16^, ^25^ In this work, 61 cases of AML‐MRC defined according to WHO 2017 criteria are studied along with 46 controls diagnosed with AML‐NOS. The aim of this study was to analyze the type and frequency of *ASXL1* mutations and their association with clinical, cytomorphological, cytogenetic, and prognostic characteristics. Also, 26 AML‐MRC patients were sequenced by next‐generation sequencing (NGS) for a panel of 19 related to AML genes.

## 2 MATERIALS AND METHODS

### 2.1 Criteria for patient selection and cytomorphological analysis

An analysis from a single center of patients diagnosed with AML‐MRC between 2008 and 2019 according to WHO 2017 criteria was performed. Sixty‐one AML‐MRC patients and a matched control group of 46 AML‐NOS diagnosed in the same period of time in the same institution were selected; cases with biallelic *CEBPA* mutations or *NPM1* mutations with multilineage dysplasia as the sole criteria for AML‐MRC were excluded.

**Figure 1:**
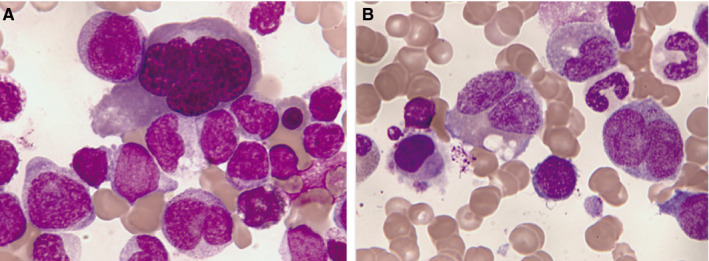
Bone marrow aspirate. May‐Grünwald‐Giemsa stain, ×1000. Acute myeloid leukemia with myelodysplasia‐related changes (AML‐MRC): A, A multinucleated giant erythroblast together with blasts and dysplastic granulocytes. B, Platelet‐forming micromegacaryocyte (on the left of the image) which is accompanied by severely dysplastic granulopoiesis

Peripheral blood and bone marrow aspirate smears performed at diagnosis and stained with May‐Grünwald‐Giemsa were reviewed independently by two expert cytologists. In the bone marrow, the percentage of dysplastic cells in each cell line was evaluated on a minimum of 25 erythroblasts (usually 100 each one), 25 neutrophils (usually 100), and 10 megakaryocytes (usually 30). The following features of dyshemopoiesis were reviewed and recorded for each lineage: erythroid lineage (internuclear bridges, nucleus lobulation, multinuclearity, karyorhexis, macrocytosis/megaloblastic changes, vacuolization, PAS positivity, and presence of ring sideroblasts in cases with PERLS staining); in granulocytes (hyposegmentation, included pseudo‐Pelger forms, hypersegmentation, mirror/ring nuclei, hypogranularity, pseudo‐Chédiak‐Higashi granules, small size, giantism); and in megakaryocytes (micromegakaryocytes, hypolobulated nuclei, separated multiple nuclei, small megakaryocytes) (Figure 1). The criteria of myelodysplasia‐related changes when based on morphology, according to WHO, were established when at least 50% of dysplastic elements in two or more cell lines were observed.

### 2.2 *ASXL1* exon 14 sequencing and NGS

*ASXL1* exon 14 was sequenced by Sanger in 61 patients with AML‐MRC and in a control group of 46 patients with AML‐NOS. The DNA was extracted from whole peripheral blood or from cultured bone marrow cells preserved in Carnoy, with Qiagen mini blood DNA kit (Qiagen) according to the manufacturer's recommendations. Sanger sequencing of exon 14 of *ASXL1* was carried out as described by Gelsi‐Boyer^17^ with modifications. The corresponding coding region from amino acid 556 to amino acid 1220, in exon 14 of the genomic sequence NG_027868 (last exon of the gene), was sequenced by Sanger and *nonsense* and *frame‐shift* mutations were searched for. The PCR reactions were carried out with Hot Start DreamTaq (Thermo Fisher) under the same thermocycler conditions as described by Gelsi‐Boyer and purified with ExoSAP (GE). Sequencing reactions were performed with BigDye kit 3.1 (Thermo Fisher) according to the manufacturer's recommendations. These were purified with SigmaSpin columns (Sigma‐Aldrich) and sequenced in a 3130xl Genetic Analyzer from Applied Biosystems.

Target sequencing of a panel including 19 AML‐related genes was performed by NGS using S5XL sequencer (Ion Torrent™). The Ion AmpliSeq™ AML Cancer Research Panel included following genes: *CEPBA, DNMT3A, GATA2, TET2, TP53, ASXL1, BRAF, CBL, FLT3, IDH1, IDH2, JAK2, KIT, KRAS, NPM1, NRAS, PTPN11, RUNX1, WT1*.

### 2.3 Statistical analysis

In order to explore clinical and cytomorphological characteristics associated to the *ASXL1* mutation in AML‐MRC, the frequencies of the categorical variables or the means of the quantitative variables in mutated AML‐MRC (*ASXL1*+) versus non‐mutated (*ASXL1*−) have been compared. Chi‐square or Fisher tests were used to compare the categorical variables in patients with and without mutations in *ASXL1.* T‐test was used to compare the means of the quantitative variables between patients with and without mutations in *ASXL1*. Survival medians were calculated according to the Kaplan‐Meier method, and compared using the log‐rank test. The Cox regression was used to estimate the hazard ratio.

## 3 RESULTS

### 3.1 Clinical and cytomorphological characteristics of patients with AML‐MRC

The main clinical characteristics of AML‐MRC patients are described in Table 1. Regarding diagnostic criteria, 25 of 61 (41%) patients had a prior diagnosis of MDS or MDS/MPN, and 49 of 61 (80%) patients presented multilineage dysplasia; of these, 17 patients were diagnosed just by morphological criteria. In bone marrow, 33 of 48 (69%) cases presented dyserythropoiesis at a percentage of ≥50%, the most frequent being the presence of cytoplasmic defects; 50 of 53 (94%) presented dysgranulopoiesis ≥50%, hypogranularity being the most frequent anomaly, and 42 of 49 (86%) cases presented megakaryocyte dysplasia ≥50%, where the most frequent anomaly was the presence of hipolobulated nuclei megakaryocytes. In 21 of 47 evaluable cases, micromegakaryocytes (45%) were observed. Regarding cytogenetics, out of 60 patients with cytogenetic assays available, 25 (42%) patients presented with normal karyotype and 24 (40%) presented cytogenetic abnormalities related to myelodysplasia, the most frequent being complex karyotype in 17 (71%) cases, loss of chromosome 7 or (−7q) in 3 (13%) cases, and loss of 5q in 2 (8%) cases (Table 1).

**Table 1:** Clinical and biological characteristics of (A) AML‐MRC patients and (B) AML‐NOS control cohort

Parameter	Value
(A) AML‐MRC patients	
Number of patients (N)	61
Age (years), mean (range)	68 (35‐89)
Male/Female	35/26
Peripheral blood	
Hemoglobin (g/l), mean (range)	85 (42‐137)
Leukocyte count (× 10^9^/l), mean (range)	15.4 (0.2‐113)
Neutrophil count (× 10e^9^/l), mean (range)	3.2 (0‐36)
Platelet count (× 10e^9^/l) mean (range)	77.2 (8‐347)
Blasts (%), mean (range)	24.2 (0‐82)
Bone marrow blasts (%), mean (range)	48.9 (7‐94)
AML de novo	36/61
AML with MDS or MDS‐MPN history	25/61
Cytogenetic	
Normal	25/60
MDS‐related cytogenetics: (N/total)	
Complex karyotype	17/60
−7/del(7q)	3/60
−5/del(5q)	2/60
del(11q)	2/60
Other abnormalities	11/60
Overall survival (OS) (median days, 95%CI)	219 (126.9‐311.1)
Follow‐up of survivals (days): mean (range), N	405 (37‐1241), 13
(B) AML‐NOS control cohort	
Number of patients (N)	46
Age (years), mean (range)	58 (17‐86)
Male/Female	20/26
Peripheral blood	
Hb (g/L), mean (range)	95.1 (66‐130)
Leukocyte count (×10^9^/l), mean (range)	68.7 (1.04‐371.8)
Platelet count (x10e^9^/l) mean (range)	84.5 (12‐353)
Blasts (%), mean (range)	46.5 (0‐99)
Bone marrow blasts (%), mean (range)	68.2 (10‐98)
Cytogenetic	
Normal	34/41
MDS‐related cytogenetics: (N/total)	
Complex karyotype	0/41
−7/del(7q)	0/41
−5/del(5q)	0/41
del(11q)	0/41
Other abnormalities	7/41
Overall survival (OS) (median days, 95%CI)	266 (69‐463)
Follow‐up of non‐*exitus* (days): mean (range), N	365 (12‐932), 19

Abbreviations: AML‐MRC, acute myeloid leukemia with myelodysplasia‐related changes; AML‐NOS, acute myeloid leukemia without other specification; MDS, myelodysplastic syndrome; MPN, myeloproliferative neoplasms.

Fifty‐two patients received treatment with either chemotherapy (n = 38) or azacytidine (N = 14), 9 patients received palliative support therapy. Nine of them underwent a hematopoietic stem cell transplantation. The median survival of the 61 cases of AML‐MRC was 219 days 95% CI (126.9‐311.1).

### 3.2 Diversity of mutations in *ASXL1*

Sequences were searched for *frameshift* mutations and *nonsense* mutations. In AML‐MRC two *nonsense* mutation (p.R693* and p.Q965*) and 9 different *frameshift* mutations were found. The most frequent mutation was p.G646fs*12 in 8 AML‐MRC patients; followed by p.E635fs*15, in 2 patients with AML‐MRC and 1 control with AML‐NOS. In addition, each of the following mutations: p.A627fs*8, p.R634fs*62, p.I641fs*15, p.G643fs*62, p.R715fs*10, p.S770fs*1, and p.L775fs*1 were found in 1 AML‐MRC patient, respectively; and p.I641fs*16 in an AML‐NOS control. The p.I641fs*15 in AML‐MRC and p.I641fs*16 in AML‐NOS mutations, were the only ones that had not been previously described in the COSMIC database.

The prevalence of *ASXL1* somatic mutations in AML‐MRC was 31% (19 of 61); significantly higher than in the 46 AML‐NOS controls, N = 2 (4.3%), *P* = .007. Additionally, several *missense* mutations were found, but they have not been considered in this work due to the inability to discern between the variants with a loss of function effect in the protein and the nonfunctional variants.

### 3.3 Mutations in other AML‐related genes

Out of 26 patients sequenced by NGS for 19 AML‐related genes, 10 of them showed *TP53* mutation and 6 of them showed *RUNX1* mutation. Other mutated genes in a lower rate were: *TET2* (5 patients), *NRAS* (5), *SRSF2* (4), *CBL* (3), *IDH2* (3), *DNMT3A* (2), *SF3B1* (2), *PTPN11* (2), *KRAS* (1), *JAK2* (1), *EZH2* (1), *CEBPA* (1 patient with complex karyotype), *CALR* (1), *U2AF1* (1), *PRPF8* (1), *ZRSR2* (1).

### 3.4 Association of mutations in exon 14 of *ASXL1* with clinical, cytomorphological and genectics characteristics in AML‐MRC

*ASXL1* mutation was associated with older age; 73.7‐year‐old ± 2.2 in *ASXL1*+ versus 65.5 ± 2.2 in *ASXL1*−, *P* = .027.

The AML‐MRC *ASXL1+ *cases were predominantly patients with a previous history of MDS or MDS/MPN: 14 of 19 (73.7%) with previous MDS or MDS/MPN in *ASXL1+ *as compared to 11 out of 42 (26.2%) in AML‐MRC *ASXL1−* patients, *P* < .001.

**Figure 2:**
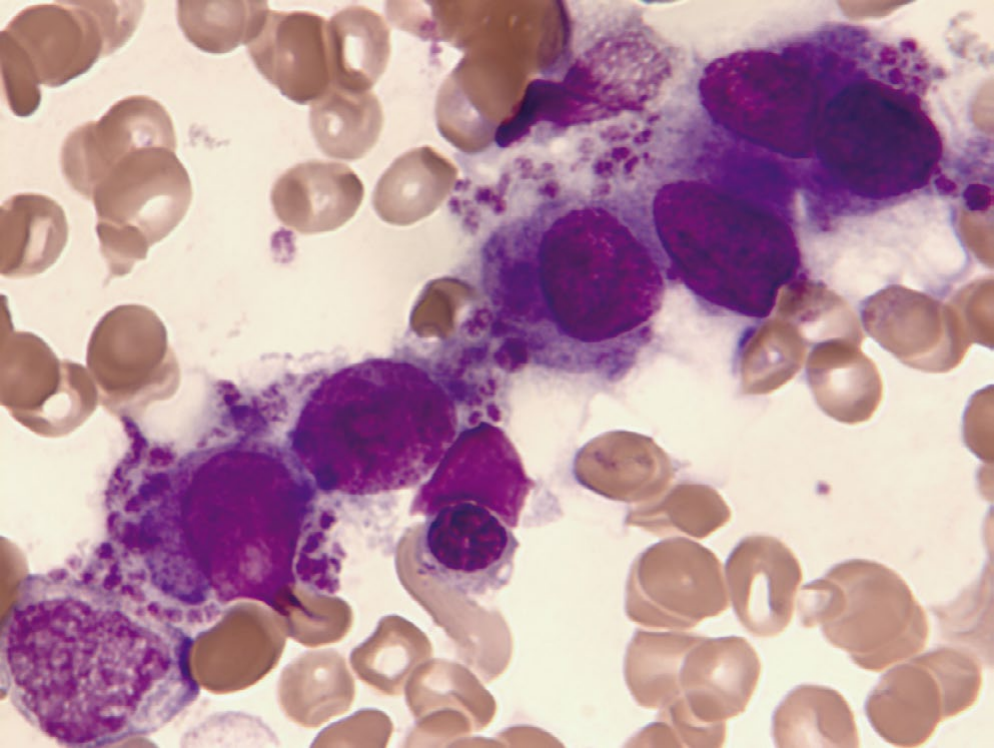
Bone marrow aspirate. May‐Grünwald‐Giemsa stain, ×1000. AML‐MRC: platelet‐forming micromegacaryocytes group

Regarding analytical and cytomorphological characteristics, differences were found in: leukocytes number (x10^9^/L mean ± SEM): 27 ± 7.2 in *ASXL1*+ versus 10.1 ± 2.2 in *ASXL1−*, *P* = .005; myelomonocytic or monocytic morphological subtypes: 12 out of 19 (63.2%) in *ASXL1+* versus 8 of 42 (19%) in *ASXL1*−, *P* = .001; percentage of blasts in bone marrow (% mean ± SEM): 38.8 ± 4.5 in *ASXL1*+ versus 53.6 ± 3.1 in *ASXL1−*, *P* = .009; and presence of micromegakaryocytes in bone marrow: 10 of 15 (66.6%) in *ASXL1*+ versus 11 of 33 (33.3%) in *ASXL1*−, *P* = .031, (Figure 2). An association trend was also observed, although without statistical significance in: presence of dismegakaryopoiesis ≥50% in bone marrow 14 of 14 (100%) in *ASXL1+* versus 28 of 35 (75.7%) in *ASXL1*−, *P* = .071) and absence of Auer rods: 1 of 19 (5.2%) with Auer rods in *ASXL1*+ versus 10 of 42 (23.8%) in *ASXL1*−, *P* = .081.

Regarding cytogenetics, an association with normal karyotype was observed (14 of 19, 74% in *ASXL1+* versus 11 of 41, 27% in *ASXL1*−, *P* = .001). In the five patients with mutation in *ASXL1*+ who did not have a normal karyotype, there were three trisomy 8, one trisomy 21, and a der(7;15)(q10;q10). Moreover, no patient with myelodysplasia‐related cytogenetic abnormalities presented mutations (0 of 19 in *ASXL1*+ vs 25 of 40, 63% in *ASXL1*−, *P* < .001).

*ASXL1* mutation showed a negative correlation with *TP53* mutation (0 of 6 in *ASXL1*+ vs 9 of 19, 47.4% in *ASXL1*−, *P* = .035), and a suggestive positive correlation with *RUNX1* mutation (3 of 6, 50% in *ASXL1*+ vs 3 of 19, 15.8% in *ASXL1*−, *P* = .087).

**Figure 3:**
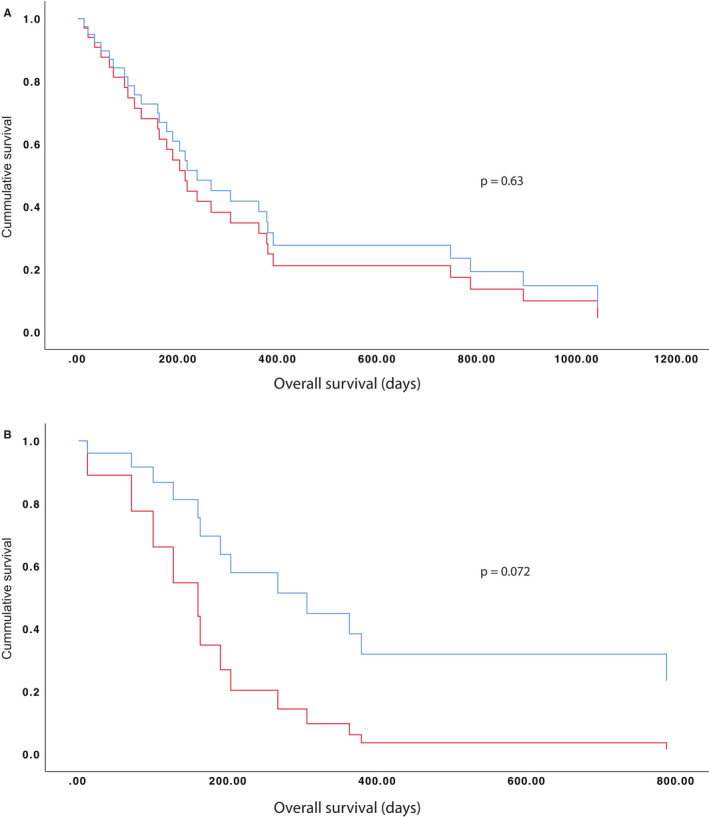
Cox regression curves of AML‐MRC patients with or without *ASXL1* mutation. A, AML‐MRC with intermediate‐risk karyotype (N = 35, *P* = .63). B, Only de novo AML‐MRC patients are included, excluding those with MDS or MDS/MPN history (N = 17, *P* = .072). Red line: *ASXL1* positive patients. Blue line: *ASXL1* negative patients

Considering AML‐MRC patients with intermediate cytogenetic prognosis (N = 35), the median survival was 178 days 95% CI (12‐344, N = 19) in *ASXL1*+, compared to 219 days CI 95% (145‐293, N = 16) in *ASXL1*−, *P* = .63 (Figure 3A). Within the group of de novo AML and intermediate risk karyotype, excluding patients with previous MDS or MDS/MPN, median survival of *ASXL1*+ was 100 days 95% CI (38‐162, N = 5) and of *ASXL1*− was 363 days 95% CI (222‐504, N = 12), *P* = .061, which represent a hazard ratio of *ASXL1* mutation of 2.9 95% CI (0.9‐9.2), *P* = .072 (Figure 3B). These differences are summarized in Table 2.

**Table 2:** Differences between *ASXL1* + and *ASXL1−* AML‐MRC patients

Parameter		*ASXL1 + *AML‐MRC	*ASXL1−*AML‐MRC	*P*‐value
Age (mean year‐old ± ESM)		73.7 ± 2.2	65.5 ± 2.2	.027
MDS or MDS/MPN history (cases+/N, %)		14/19 (73.7%)	11/42 (26.2%)	<.001
Leukocyte count (×10^9^/l mean ± ESM)		27 ± 7.2	10.1 ± 2.2	.005
Morphological features (monocytic/myelomonocytic) (cases+/N, %)		12/19 (63.2%)	8/42 (19%)	.001
BM blasts (% mean ± ESM)		38.8 ± 4.5	53.6 ± 3.1	.009
Auer rods (cases+/N, %)		1/19 (5.2%)	10/42 (23.8%)	.081
BM micromegakaryocytes (cases+/N)		10/15 (66.6%)	11/33 (33.3%)	.031
BM dismegakaryopoyesis ≥50% (cases+/N, %)		14/14 (100%)	28/35 (75.7%)	.071
*RUNX1*(mutated cases/N)		3/6 (50%)	3/19 (15.8%)	.087
*TP53* (mutated cases/N)		0/6 (0%)	9/19 (47.4%)	.035
Normal karyotype (N, %)		14/19 (74%)	11/41 (27%)	.001
MDS‐related cytogenetic abnormality (N, %)		0/19 (0%)	25/40 (63%)	<.001
Overall survival in de novo AML‐MRC intermediate‐risk karyotype	Median, 95% CI	100 (38‐162)	363 (222‐504)	.061
		N = 5	N = 12	
	HR, 95% CI	2.9 (0.9‐9.2)		.072

Abbreviations: AML‐MRC, acute myeloid leukemia with myelodysplasia‐related changes; BM, bone marrow; MDS, myelodysplastic syndrome; MPN, myeloproliferative neoplasms.

### 3.5 Characteristics of patients with AML‐NOS with *ASXL1* mutation

Only two male patients within the AML‐NOS group had a mutation in *ASXL1* who were 80 and 86 years old, respectively. One had a leukocyte count of 30.9 × 10^9^/L, while the other had 26.8 × 10^9^/L. Both had been diagnosed with myelomonocytic AML according to cytomorphological criteria. They also shared a severe dysgranulopoiesis (quantitatively >50%) in bone marrow, and the erythroid and megakaryocytic series dysplasia could not be assessed as they were greatly reduced at diagnosis. One of the cases presented normal karyotype and the second displayed trisomy 8 and 13. The mean survival of these *ASXL1+ *patients was 21 days, 95% CI (20‐22) compared to 909 days of *ASXL1−* patients in this group, 95% CI (590‐1228) (*P* = .01), HR 6.25 (1.25‐31.25), *P* = .025.

The characteristics of patients with AML‐MRC and AML‐NOS with mutation in *ASXL1* are summarized in the Table 3.

**Table 3:** List of patients (AML‐MRC) and controls (AML‐NOS) harboring *ASXL*1 mutations, ordered by mutated amino acid position

ID	AA change	cDNA change	DIAGNOSIS	COSMIC ID	AGE	MDS‐MDS/MPN HISTORY	Monocytic/myelomonocytic features	Karyotype	Therapy	Overall survival (days)
1	p.A627fs*8	c.1879_1880insG	AML‐MRC	COSM1012908	77	YES	YES	46,XX[20]	A	1043
2	p.R634fs*62	c.1900_1921del22	AML‐MRC	COSM219102	68	NO	NO	47,XY,+8[6]/48,idem,+9[3]/46,XY[11]	F	12
3	p.I641fs*16	c.1922insA	AML‐NOS	Unreported	80	NO	YES	46,XY [20]	F	20
4	p.I641fs*15	c.1923_1927del5	AML‐MRC	Unreported	67	NO	YES	46,XX[20]	F	100
5	p.E635fs*15	c.1888_1910del23	AML‐MRC	COSM51200	58	YES	YES	46,XY[20]	A	33
6	p.E635fs*15	c.1899_1921del23	AML‐MRC	COSM41597	81	YES	NO	46,XY[20]	A	894
7	p.E635fs*15	c.1899_1921del23	AML‐NOS	COSM41597	86	NO	YES	48,XY,+8,+13[18]/46,XY[2]	P	21
8	p.G643fs*15	c.1926dup	AML‐MRC	COSM4385691	75	YES	NO	47,XY,+8[16]/46,XY[4]	A	86
9	p.G646fs*12	c.1934_1935insG	AML‐MRC	COSM34210	86	YES	YES	46,XY[20]	C	46
10	p.G646fs*12	c.1934_1935insG	AML‐MRC	COSM34210	78	NO	NO	46,XX[20]	P	71
11	p.G646fs*12	c.1934_1935insG	AML‐MRC	COSM34210	74	YES	NO	46,XY[20]	A	381
12	p.G646fs*12	c.1934_1935insG	AML‐MRC	COSM34210	78	YES	YES	46,XY[20]	A	748
13	p.G646fs*12	c.1934_1935insG	AML‐MRC	COSM34210	73	YES	YES	46,XX[20]	A	385
14	p.G646fs*12	c.1934_1935insG	AML‐MRC	COSM34210	49	YES	YES	46,XY[20]	I + C	239
15	p.G646fs*12	c.1934_1935insG	AML‐MRC	COSM34210	75	NO	YES	45,XX,der(7;15)(q10;q10)[5]/46,XX[5]	C	788
16	p.G646fs*12	c.1934_1935insG	AML‐MRC	COSM34210	89	NO	NO	46,XY[14]	P	127
17	p.R693*	c.2077C > T	AML‐MRC	COSM51388	71	YES	YES	46,XY[20]	A	178
18	p.R715fs*10	c.2141delC	AML‐MRC	COSM3719373	87	YES	NO	46,XY[20]	A	113
19	p.S770fs*1	c.2309_2309delC	AML‐MRC	COSM5944120	70	YES	YES	46,XX,+8[20]	A	93
20	p.L775fs*1	c.2324_2324delT	AML‐MRC	COSM53206	79	YES	YES	47,XY,+21[18]/46,XY[2]	A	392
21	p.R965*	c.2893C > T	AML‐MRC	COSM267971	73	YES	YES	46,XY[20]	C	37

Abbreviations: A, Azacytidine; AA, Amino acid; BM, Bone Marrow; AML‐MRC, acute myeloid leukemia with myelodysplasia‐related changes; AML‐NOS, acute myeloid leukemia without other specifications; C, Ara‐C; F, FLUGA, I, Idarubicin; MDS, Myelodysplastic Syndrome; MPN, Myeloproliferative Neoplasm; NE, Not Evaluable; P, Palliative.

## 4 DISCUSSION

*Nonsense* and *frameshift ASXL1* mutations in exon 14 produce a truncated protein in the C‐terminal PHD domain. These loss‐of‐function mutations have been associated with different myeloid disorders, participating in leukemogenesis and, in general, contributing to a worse prognosis. The globally described frequency in AML is 10.8%‐14.5%,^16^, ^26^, ^27^, ^28^, ^29^, ^30^ with the highest incidence being reported in AML‐MRC: 20.8%‐35%.^15^, ^16^ The search for clinic‐biological characteristics associated with the presence of this mutation in AML has been attempted in some studies with variable results, partly possibly due to the very different patient cohorts evaluated.^16^, ^26^, ^28^ In this series, we focused on the study of *ASXL1* mutations in patients with AML‐MRC defined according to the latest WHO update, namely excluding patients with *NPM1* mutation and *CEBPA* biallelic mutation, as opposed to previous studies. The results obtained confirm a high frequency of *ASXL1* mutations in patients with AML‐MRC (31%), compared to a control group of patients with AML‐NOS (4.3%). With regard to the type of mutations found, most of them are *frameshift* (19 patients), while only two cases are *nonsense* mutations. Only two of these mutations have not been previously described in the COSMIC database (p.I641fs*15 and p.I641fs*16). The most frequent mutation is p.G646fs*12, as in previously published series on myeloid pathology**.** 31

When comparing the clinical, cytomorphological, and cytogenetic characteristics of patients with AML‐MRC *ASXL1+ *versus *ASXL1−,* significant differences are found. Within the AML‐MRC, *ASXL1* mutations are mainly associated with older age and a background of MDS/MDS‐MPN cases. Although this last association had been reported in some AML *ASXL1+ *series,^24^, ^28^, ^29^ other publications, focused on patients with AML‐MRC, had shown contradictory results.^15^, ^16^ In MDS and CMML the presence of *ASXL1* mutations have been associated with shorter transformation time to AML,^19^, ^20^ which could partly support the association of this mutation with a history of MDS and MDS‐MPN.

In the current study, *ASXL1+ *patients have a higher leukocyte count at diagnosis as compared with nonmutated patients. No differences were found regarding the hemoglobin and platelets levels between both groups. We highlight the morphological differences in AML‐MRC *ASXL1+ *patients compared to *ASXL1−*, such as the presence of a higher frequency of micromegakaryocytes in bone marrow. In addition, *ASXL1+ *patients showed a trend toward a higher presence of megakaryocyte dysplasia (≥50%) in bone marrow. While the association between presence of mutated *ASXL1* in AML‐MRC and a higher frequency of dysgranulopoiesis has already been reported,^16^ we have not found references regarding the association of this mutation with specific signs of dysplasia, such as the presence of micromegakaryocytes. Despite the number of patients in our series is limited, the higher presence of some morphological signs of dysplasia in *ASXL1+ *versus *ASXL1−* patients would support the role of this mutation as a possible dysplasia‐associated molecular marker.^16^, ^32^ With regard to cytogenetics, the mutation in *ASXL1* has previously been associated with the absence of cytogenetic abnormalities related to myelodysplasia and intermediate cytogenetic risk in patients with AML‐MRC.^16^ In addition, *ASXL* mutations have been associated with various abnormalities such as trisomy 8 and alterations on chromosome 11 in all AMLs.^26^, ^28^ In our series, we confirmed an association of *ASXL1* mutation with normal karyotype; up to 56% of patients with AML‐MRC displaying a normal karyotype are *ASXL1+*. Of the five *ASXL1+ *patients who had cytogenetic abnormalities, three are trisomy 8. No *ASXL1+ *cases presented complex karyotypes or other myelodysplastic syndrome‐related cytogenetic abnormalities, thus suggesting that both findings might be mutually exclusive and redundant from a pathophysiological point of view. Also the *ASXL1* mutation showed a negative correlation with the *TP53* mutation.

Differences in terms of overall survival between AML‐MRC *ASXL1+ *versus *ASXL1−* patients were found only in de novo AML‐MRC patients with intermediate‐risk karyotype (excluding MDS and MDS/MPN history and adverse‐risk karyotype), having *ASXL1*+ patients a worst outcome with a clear trend toward statistical association than *ASXL1*−, HR = 2.9 *P* = .072 (Figure 3B). On the other hand, in patients with MDS or MDS/MPN history *ASXL1* mutation did not showed a prognostic significance. Devillier et al. has also showed a negative impact on survival of the *ASXL1* mutation in AML‐MRC patients,^16^ but in our series, patients with MDS history are not significantly affected by *ASXL1* mutation. Remarkably, in the current study, unlike Devillier et al.’s, patients with *NPM1* and biallelic *CEBPA* mutation, in the absence of cytogenetic abnormalities of AML‐MRC are excluded from the group, following the latest WHO update. These are patients with a favorable prognosis which are usually *ASXL1−*. Besides, a higher patient number may be needed for the demonstration of these differences.

Finally, and although there are only two *ASXL1+ *patients in the AML‐NOS control group, we observe some common characteristics with AML‐MRC patients with *ASXL1* mutation, highlighting the presence of leukocytosis in both and myelomonocytic morphological subtype. The two patients with AML‐NOS *ASXL1+ *share a severe dysgranulopoiesis in the bone marrow aspirate, and dysplasia in the other hematopoietic lines cannot be assessed because they are scarcely represented at diagnosis. Both patients had poor survival. All above raises the possibility that these two AML‐NOS patients could be miscategorized, actually being AML‐MRC.

In conclusion, the mutation in *ASXL1* is frequent in patients with AML‐MRC and it is associated to specific features, including morphological signs of dysplasia, which could anticipate the *ASXL1* mutational status. This association and its high frequency in AML‐MRC raises the possible role of *ASXL1* as a surrogated molecular marker, which could facilitate the diagnosis of patients within this group, especially in the absence of other diagnostic criteria such as cytogenetic features, or a previous history of MDS or MDS/MPN; or when the assessment of multilineage dysplasia is morphologically difficult. This mutation could be used as a worst outcome marker in de novo AML‐MRC with intermediate‐risk karyotype. Larger studies are needed to confirm the possible role of *ASXL1* in the biological characterization of patients with AML‐MRC and to assess whether it implies a worse prognosis within this group.

## CONFLICT OF INTEREST

There are no conflicting interests.

## AUTHOR CONTRIBUTIONS

CPM: Experimental design, acquisition, analysis and interpretation of data, manuscript preparation, critical revision for intellectual content. SBS: *ASXL1* Sanger sequencing, acquisition, analysis and interpretation of data, manuscript preparation, critical revision for intellectual content. RMC: Acquisition, analysis and interpretation of data, manuscript preparation collaboration. OPL: Clinical data collection, data analysis. M. S.: Clinical data collection, data analysis. MTV: Samples submission, manuscript preparation collaboration. ECC: Samples submission, manuscript preparation collaboration. JG: Samples submission, clinical data collection. RB: Experimental design, acquisition, analysis and interpretation of data, manuscript preparation collaboration. JAPS: Experimental design, analysis and interpretation of data, critical revision for intellectual content. All authors critically reviewed and approved the manuscript for submission.

## COMPLIANCE WITH ETHICAL STANDARDS

All procedures performed in this study were in accordance with the ethical standards of the institutional research committee and with the Helsinki Declaration.

## Data Availability

The data that support the findings of this study are available from the corresponding author upon reasonable request.
